# Effect of exonic splicing regulation on synonymous codon usage in alternatively spliced exons of *Dscam*

**DOI:** 10.1186/1471-2148-9-214

**Published:** 2009-08-27

**Authors:** Aya Takahashi

**Affiliations:** 1 Division of Population Genetics, National Institute of Genetics, Mishima 411-8540, Japan; 2 Department of Genetics, Graduate University for Advanced Studies (Sokendai), Mishima 411-8540, Japan

## Abstract

**Background:**

Synonymous codon usage is typically biased towards translationally superior codons in many organisms. In *Drosophila*, genomic data indicates that translationally optimal codons and splice optimal codons are mostly mutually exclusive, and adaptation to translational efficiency is reduced in the intron-exon boundary regions where potential exonic splicing enhancers (ESEs) reside. In contrast to genomic scale analyses on large datasets, a refined study on a well-controlled set of samples can be effective in demonstrating the effects of particular splice-related factors. *Down syndrome cell adhesion molecule *(*Dscam*) has the largest number of alternatively spliced exons (ASEs) known to date, and the splicing frequency of each ASE is accessible from the relative abundance of the transcript. Thus, these ASEs comprise a unique model system for studying the effect of splicing regulation on synonymous codon usage.

**Results:**

Codon Bias Indices (CBI) in the 3' boundary regions were reduced compared to the rest of the exonic regions among 48 and 33 ASEs of exon 6 and 9 clusters, respectively. These regional differences in CBI were affected by splicing frequency and distance from adjacent exons. Synonymous divergence levels between the 3' boundary region and the remaining exonic region of exon 6 ASEs were similar. Additionally, another sensitive comparison of paralogous exonic regions in recently retrotransposed processed genes and their parental genes revealed that, in the former, the differences in CBI between what were formerly the central regions and the boundary regions gradually became smaller over time.

**Conclusion:**

Analyses of the multiple ASEs of *Dscam *allowed direct tests of the effect of splice-related factors on synonymous codon usage and provided clear evidence that synonymous codon usage bias is restricted by exonic splicing signals near the intron-exon boundary. A similar synonymous divergence level between the different exonic regions suggests that the intensity of splice-related selection is generally weak and comparable to that of translational selection. Finally, the leveling off of differences in codon bias over time in retrotransposed genes meets the direct prediction of the tradeoff model that invokes conflict between translational superiority and splicing regulation, and strengthens the conclusions obtained from *Dscam*.

## Background

Genomic data from many different organisms indicate that synonymous codon usage is typically biased. This bias is an intriguing property that reflects a composite of different evolutionary forces such as mutational bias, genetic drift, and natural selection [1-3]. In many species, including *Drosophila*, one of the dominant forces affecting the bias is thought to be natural selection for translational efficiency or accuracy, or both (reviewed in [4]). The most abundant codons are preferred because of the abundance of the cognate tRNAs [5-9], and thus the use of the codons maximizes the translation speed and minimizes the amino acid misincorporation rate [3,10]. There is limited experimental evidence that major codons are translationally superior [11-14], but empirical data can be explained well in this framework.

The selective force for translational efficiency is weak enough to allow confounding neutral processes to dominate in some cases [15]. For example, the effect of genetic drift (population size) is inferred from the comparison between *D. melanogaster *and *D. simulans *[16,17]. Other types of selective forces may also counteract translational selection at synonymous sites. Splicing regulation is one of them. Specifically, there are binding sites within exonic regions for serine-arginine-rich (SR) proteins, which enable correct splicing. These binding sites, known as exonic splicing enhancers (ESEs), are usually located within the vicinity of potential intron-exon junctions [18], and many of them are ~6–15 bp in length [19]. These splice signals within the coding regions can potentially affect synonymous codon usage. Recent genomic scale analyses in *D. melanogaster *revealed that translationally optimum codons are not preferred near the intron-exon boundary, and thus are not splice optima [20]. Moreover, Warnecke and Hurst [20] showed that codons putatively involved in ESEs are almost never translationally optimal, and therefore, a conflict exists between translational advantage and splice efficiency in codon usage. They also showed that this conflict is larger in highly expressed genes, although the effect of expression level was not very strong. These conclusions obtained from a large genomic scale dataset stimulated me to find a well-controlled gene system in which splicing regulation can be effectively analyzed.

**Figure 1:**
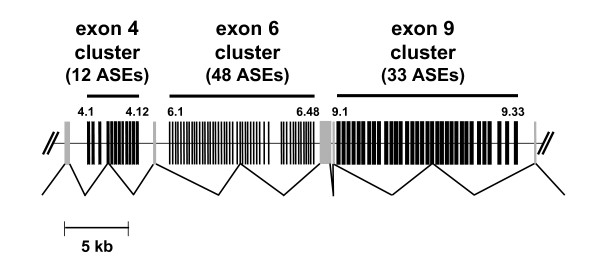
**Partial structure of *Down syndrome cell adhesion molecule *(*Dscam*) in *D. melanogaster***. Black and gray vertical bars represent mutually exclusive alternatively spliced exons (ASEs) and constitutive exons, respectively. The exon 4, exon 6, and exon 9 clusters consist of 12 (exon 4.1–4.12), 48 (exon 6.1–6.48), and 33 (exon 9.1–9.33) mutually exclusive ASEs, respectively. These ASE clusters are indicated by horizontal bars above the diagram.

Alternatively spliced exons (ASEs) of *Down syndrome cell adhesion molecule *(*Dscam*) appeared to be an excellent model system to study such regulation, because this molecule contains the largest number of ASEs known to date. There are four exons that have multiple alternatively spliced forms (12, 48, 33, and 2 forms, for exons 4, 6, 9, and 17, respectively), which produces 38,016 distinct potential isoforms [21]. The structure of the gene is shown in Figure 1. These ASEs can generate unique cell identity by expressing distinct sets of isoforms in the nervous systems where the molecule plays a crucial role in neuronal wiring (reviewed in [22]). Also, the large spectrum of distinct isoforms is required in hemocytes where the molecule is necessary for effective immune response [23]. Thus, this molecule's unique gene structure has probably arisen from the necessity to generate a diverse repertoire of alternatively spliced variants.

In addition to the fact that *Dscam *contains the largest number of ASEs known to date, these ASEs provide an ideal system for analyzing the effect of splicing regulation for the following reasons. First of all, these ASEs are spliced out from a single pre-mRNA, and therefore, splicing frequency can be separated from transcription frequency. This kind of within-transcript comparison is the only way to analyze the effect of splicing frequency. These ASEs are also under the control of the same splicing machinery within the same cell environment in which they are expressed. Second, since these ASEs are in the same genomic region, they are controlled for many possible confounding factors affecting synonymous codon usage such as local GC content [24-26] and recombination rate [27-31]. Finally, these ASEs are similar in length and amino acid composition [21], which are also factors known to influence the codon usage [28,32]. It should be noted that most of these paralogous ASEs diverged prior to the *D. melanogaster *– *D. virilis *species split [33], which indicates that the synonymous sites of these ASEs have diverged beyond saturation [34,35]. Therefore, it is reasonable to assume that their codon usage is effectively independent from each other. Additionally, all the 12 species of *Drosophila *whose genomic sequences are available have diverged after the *D. melanogaster *– *D. virilis *species split [36], and thus, orthologous pairs of ASEs can be identified in many cases.

In this study, I analyzed these ASEs to delineate the effect of splicing regulation on synonymous codon usage by comparing the levels of codon bias in the central and intron-exon boundary regions of these exons. I studied the effect of two splice-related factors that affect the regional differences in CBI among these ASEs; the splicing frequency (expression level) and the distance from adjacent constitutive exons. In addition, I also conducted a comparison between paralogous exonic regions of retrotransposed processed genes and their parental genes, in order to investigate the effect of splicing regulation on synonymous codon usage in different gene systems.

## Results and discussion

### Codon usage bias towards translationally superior codons is reduced near the 3' intron-exon boundaries in most of the *Dscam *exons

Genome-wide analysis in *Drosophila *has indicated reduced codon usage bias toward translationally optimal codons within 48 nucleotides of an intron-exon boundary, where the vast majority of functional ESEs are assumed to exist [18,20]. In order to investigate whether this trend exists in the ASEs of *Dscam *exons 4, 6, and 9, I calculated Codon Bias Index (CBI) in the center and the boundary regions and compared the differences. Following Warnecke and Hurst [20], the boundary region was defined as the exonic region within 15 full codons of the boundary, excluding the partial codons at the junction, and the center region was defined as the regions remaining after subtracting the flanks (22–26 codons for exon 4, 8–13 codons for exon 6, 62–71 codons for exon 9).

**Figure 2:**
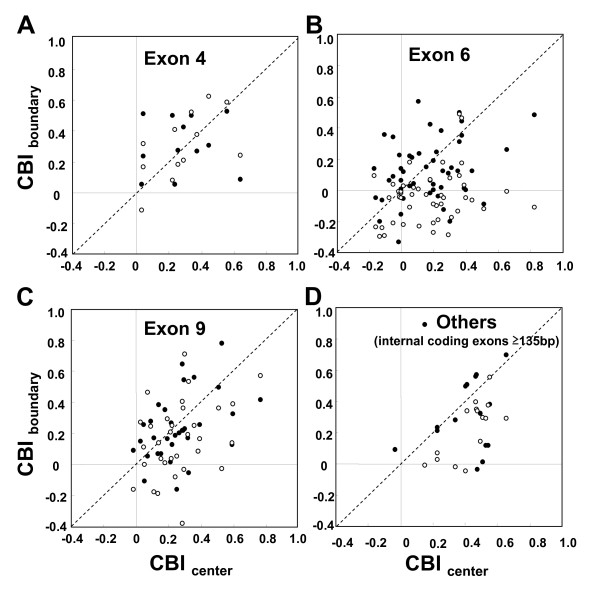
**Regional differences in codon usage pattern among ASEs of *Dscam***. Relationship between the codon bias indices (CBI) of the center and the intron-exon boundary regions (15 full codons from the junction) among the ASEs of *D. melanogaster Dscam *exon 4 (A), among those of exon 6 (B), and among those of exon 9 (C). Those among other internal coding exons longer than 135 bp (exon 3, 5, 7, 8, 11–16, 17.1, 17.2, 18–22) are also shown (D). Closed and open circles indicate 5' and 3' boundary regions, respectively.

Comparisons of CBI values between the center and the boundary regions of these exon clusters are shown in Figure 2. CBI values of the 3' boundary region are lower than the central exonic regions in the ASEs of exons 6 and 9 (Figure 2B and 2C, respectively). Among the exon 6 ASEs (N = 48), there are significant differences between the center and 3' boundary (*z *= -4.94, *P *< 0.001, Wilcoxon matched-pair signed rank test; the same below, except where indicated) and 5' boundary and 3' boundary (*z *= -4.45, *P *< 0.001), but not between center and 5' boundary (*z *= -1.272, *P *= 0.20). Among the exon 9 ASEs (N = 33), the difference between center and 3' boundary is significant (*z *= -2.44, *P *< 0.05), but the differences between center and 5' boundary (*z *= -0.94, *P *= 0.34), and 5' boundary and 3' boundary (*z *= -1.51, *P *= 0.13) are not significant. Other internal exons which are longer than 135 bp (N = 16) also show significant differences between the center and 3' boundary (*z *= -3.46, *P *< 0.001), the center and 5' boundary (*z *= -2.07, *P *< 0.05), and 5' boundary and 3' boundary (*z *= -2.43, *P *< 0.05). These comparisons indicate that in most of the exons in this gene, codons in the 3' boundary regions are less adapted for translation efficiency compared to codons in the central exonic regions.

Since the regions analyzed were short, the reduction of CBI in the 3' boundary region could be due to bias in amino acid composition. I compared the codon usage pattern of each amino acid in the 3' boundary region and the remaining 5' region in a pooled sample of exon 6 and 9 ASEs. Among 18 amino acids with degenerate codons, 16 showed higher frequencies of translationally preferred codons in the remaining region (5' boundary plus central) compared to those in the 3' boundary region (see Additional file 1). Among them, 7 amino acids (7/18 = 39%) showed statistical significance at the level of *P *< 0.05 by Fisher's exact test. These results clearly indicate that the bias in amino acid composition is not the reason for the reduced CBI in the 3' boundary regions.

Since Warnecke and Hurst [20] indicated that translationally optimal codons and splice optimal codons (potential ESEs) in *D. melanogaster *are mostly mutually exclusive, my data suggest stronger restriction on codon usage due to splicing regulation in the 3' boundary regions than in the 5' boundary regions throughout the gene, except in the ASEs of the exon 4 cluster. Despite the genome-wide tendency of reduced CBI in the intron-exon boundary regions [20], various patterns among different genes and exons can arise at least partly by different machineries used for splicing regulation (reviewed in [37-39]). For example, Olson et al. [40] have shown that the heterogeneous nuclear ribosomal protein hrp36 plays a key role as a splicing repressor in mutually exclusive splicing of the *Dscam *exon 6 ASEs, but depletion of this protein had no effect on the splicing of ASEs in other exon clusters. A possible reason for the distinct codon usage pattern in the 3' boundary regions could be that some of the splicing machineries used in this gene utilize signals in these regions to a larger extent than those in other exonic regions.

Specifically, the reduced CBI_3' boundary region_in the ASEs of the exon 6 cluster, where the difference in CBI between the center and the 3' boundary regions is most apparent (Figure 2), may be explained by the unique model for the mechanism of mutually exclusive alternative splicing proposed by Graveley [41]. He discovered two classes of conserved elements in the non-coding regions of the *Dscam *exon 6 cluster; the docking site, located in the intron downstream of constitutive exon 5, and the selector sequences, which are located upstream of each exon 6 ASE. Each selector sequence is complementary to a portion of the docking site. The model suggests that the formation of the RNA structure by docking site:selector sequence interaction is a central component of the mechanism guaranteeing that only one exon 6 ASE is included in each *Dscam *mRNA. This docking site:selector site interaction brings together the 3' end of exon 5 and the 5' end of one of the exon 6 ASEs. If this is the case, there must be another factor that brings together the 3' end of the chosen ASE and the 5' end of exon 7. The upstream splice reaction aided by docking site:selector site interaction and the downstream reaction may occur simultaneously, but the latter may be where ESEs are mainly involved. If so, then this could be the reason for observing stronger restriction on the codon usage in the 3' boundary regions of these exon 6 ASEs.

Although obtaining a detailed picture of the splicing machineries requires further experiments, the reduction of CBI in the 3' intron-exon boundary regions in this gene suggests that splice-related selection is present. I further examined this possibility by comparing the level of CBI reduction and experimentally obtained splicing frequency data.

### Conflict between translational selection and selection for splice efficiency increases with the splicing frequency

Iida and Akashi [42] have shown that within alternatively spliced genes, GC-ending codons are more abundant in constitutive than in alternatively spliced exons in *Drosophila *and humans. This is consistent with the prediction that translational selection should act more strongly to bias codon usage in constitutive than in alternative exon codons because the former are translated more often than the latter. The selection coefficient for translational selection is expected to become larger in the more frequently translated codons. This is also the pattern that was observed in *Dscam *when whole exonic regions of constitutive and alternative exons were compared; average CBI of 17 internal constitutive exons without UTRs was 0.344 ± 0.030 (S.E.), whereas that of 95 ASEs was 0.167 ± 0.019 (*t *= 3.96, *df *= 110, *P *< 0.001).

As suggested from the comparison of CBI between constitutive and alternative exonic regions, if majority of the *Dscam *exonic region is under translational selection codon usage in the 3' boundary regions and the remaining exonic regions should show different trends in relation to the expression level, because of the additional constraint due to splicing regulation in the former. If the latter is relatively free from the constraint due to splicing regulation, its CBI should show a positive correlation with the expression level due to translational selection. This can be the background level of translational selection. Whereas, if synonymous sites in the 3' boundary region are under selection for splicing efficiency, it is expected that CBI in this region should show relatively constant values or a negative correlation with the splicing frequency (expression level). The level of conflict, which can be evaluated by the difference between the 3' boundary region and the rest of the region (Δ-CBI_rest – 3' boundary_) should then increase with the splicing frequency.

The expression levels of multiple *Dscam *splice variants have been studied extensively by Neves et al. [43] using their custom microarray. Taking advantage of the availability of their data, I analyzed the 3' boundary region of the ASEs in exons 6 and 9, where CBI was significantly reduced in comparison to the remaining 5' region (Figure 2). I used the relative order of the expression level of each exon 6 and 9 ASE in hemocyte-derived S2 cell lines as the estimated order of splicing frequency (see Methods).

**Figure 3:**
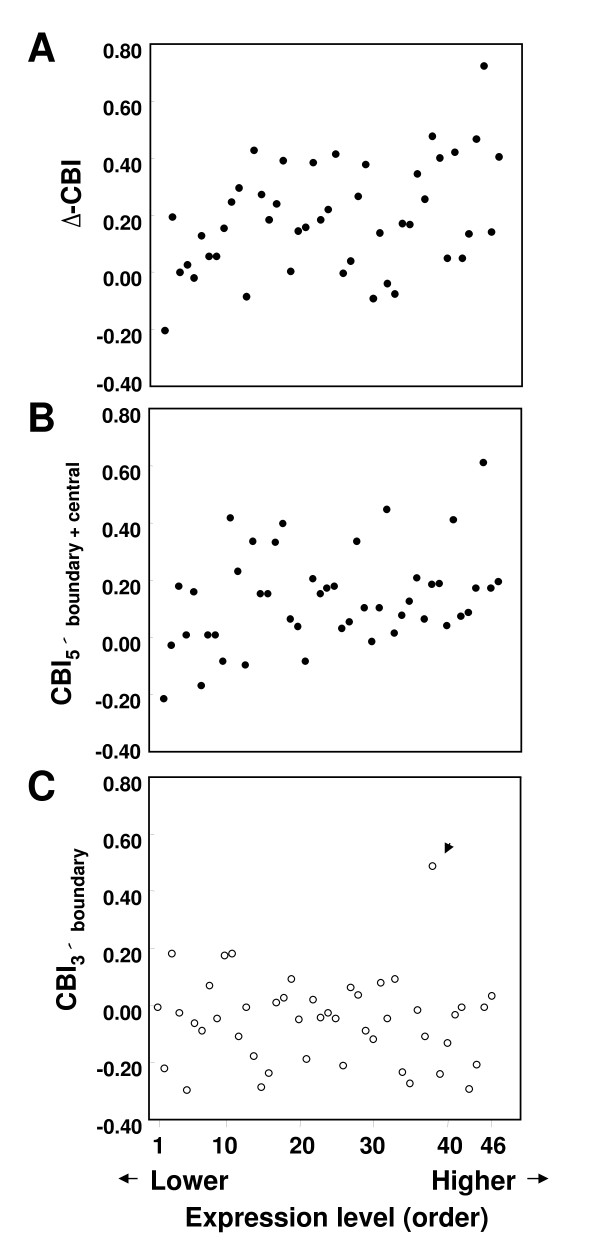
**Relationship between CBI and expression level in ASEs of *Dscam *exon 6**. Relationship between codon usage and the relative order of expression level (splicing frequency) of the *D. melanogaster Dscam *exon 6 ASEs in hemocyte-derived S2 cell lines [43]. Relationship between the expression level and the difference in CBI between the 5' intron-exon boundary plus central regions and the 3' intron-exon boundary regions (Δ-CBI_rest – 3'boundary_; A), that between the expression level and CBI in the 5' intron-exon boundary plus central regions (CBI_5'boundary + central_; B), and that between the expression level and CBI in the 3' intron-exon boundary regions (CBI_3'boundary_; C). A positive correlation was detected between the expression level and Δ-CBI_rest – 3'boundary _(*r *= 0.33, *P *< 0.05, Spearman's rank order correlation; *N *= 46), and between the expression level and CBI_5'boundary + central _(*r *= 0.34, *P *< 0.05, Spearman's rank order correlation; *N *= 46). No significant correlation was detected between the expression level and CBI_3'boundary _(*r *= -0.11, *P *= 0.45, Spearman's rank order correlation; *N *= 46). Arrowhead indicates an outlier > 3 × S. D.

In the case of exon 6, most ASEs are expressed at some or all stages of development, and their expression levels showed only moderate differences among different tissues and among different developmental stages [43]. Thus, I used all the 46 ASEs with the expression level data (data for 2 ASEs were missing due to technical issues) for the following analyses. As expected, a positive correlation was detected between the expression level and Δ-CBI_rest – 3' boundary _= CBI_5' boundary + central _– CBI_3' boundary _(Figure 3A; *r *= 0.33, *P *< 0.05, Spearman's rank order correlation; *N *= 46), and also between the expression level and the CBI of the remaining 5' region (Figure 3B; *r *= 0.34, *P *< 0.05, Spearman's rank order correlation; *N *= 46). The correlation between the CBI_3' boundary _and the splicing frequency was not significant (Figure 3C; *r *= -0.11, *P *= 0.45, Spearman's rank order correlation; *N *= 46), suggesting a relatively constant level of CBI values in this region with a weak trend of negative correlation with the splicing frequency (see Additional file 2).

The contrasting expression-level dependency of codon usage between the 3' boundary and the remaining exonic regions indicates regional differences in the type of selection affecting the synonymous codon usage within exons of these exon 6 ASEs. In particular, the positive correlation between Δ-CBI_rest – 3' boundary _and the expression level indicates that the intensity of splice-related selection counteracting the translational selection increases with the splicing frequency. Therefore, the conflict between these two evolutionary forces in the 3' boundary region becomes more pronounced in the highly expressed (spliced) ASEs of the exon 6 cluster.

In the case of exon 9 ASEs, 4 or 5 of the 33 ASEs are spliced more frequently than others in a number of tissues [23,43]. About half of the ASEs are represented at less than the overrepresentation level of 0.1 (10% expression level of the random expectation) in these tissues [43]. Since the expression level order of these less abundant ASEs is likely to be unreliable, I used only the last half of the ASEs ordered from the lowest to the highest expression level, where the actual trend in transcript abundance is visible from the heatmap (Figure four of [43]). Using these 16 ASEs of exon 9, a positive correlation between the expression level and Δ-CBI_rest – 3' boundary _(see Additional file 3 panel A; *r *= 0.54, *P *< 0.05, Spearman's rank order correlation; *N *= 16) was detected. There was no significant correlation between the expression level and CBI of the 3' boundary region (see Additional file 3 panel C; *r *= -0.24, *P *= 0.36, Spearman's rank order correlation; *N *= 16) or between the expression level and CBI of the remaining 5' region (see Additional file 3 panel B; *r *= 0.33, *P *= 0.22, Spearman's rank order correlation; *N *= 16). These results indicate that even with a smaller number of ASEs analyzed compared to the exon 6 cluster, the intensity of conflict in the exon 9 ASEs between selection for translational efficiency and for splicing efficiency also correlates with the expression level.

### The distance to the adjacent constitutive exon affects codon usage in the intron-exon boundary region

Among the mutually exclusive ASEs of this gene, the nucleotide distance to the adjacent constitutive exons is a factor, which is likely to affect the occurrence of splicing. Presumably, closer splice sites are more likely to interact. This distance effect in ASEs is analogous to the effect of intron length in the constitutive exons in humans and mouse, in which exons flanked by longer introns contained a significantly higher abundance of putative ESEs than those flanked by shorter introns [44]. This is consistent with the "exon definition" model of splicing that invokes ESE oriented initial splice-site recognition [45]. This model applies better to genes with small exons and large introns, which fits the case of exon 6 and 9 ASEs. The exon 6 ASEs spread over a ~13 kb region and the distance between splice sites varies between ~400 bp to ~10 kb. Despite this wide distance range, there was no apparent relationship between the position of ASEs relative to adjacent constitutive exons and the expression level (data not shown), which suggest that the distance effect is controlled in some way, possibly by exonic signals. It can be inferred from the docking site:selector site model that the downstream splice reaction could be the major function of the ESEs in the exon 6 ASEs. If so, the intensity of these splice signals should increase in the ASEs closer to the upstream adjacent exon 5 to complement the distance effect.

Inconsistent with this prediction, there was no significant positive correlation between CBI_3' boundary _and the sequence distance from exon 5 (*r *= 0.16, *P *= 0.28 Spearman's rank order correlation; *N *= 48). However, since the intensity of splicing regulation on synonymous codon usage is likely to vary with the position of amino acid residues, specific codons were examined to see if there are variations in codon usage that may contribute to complementing the distance effect. Four conserved amino acid residues among all the ASEs were chosen to control for the codon sequence and the position effect (see Additional file 4). The exon 6 ASEs were then divided into 3 groups according to the proximity to exon 5, and the number of translationally preferred and unpreferred codons used in these groups for each conserved amino acid residue were counted (see Additional file 5). The frequencies of unpreferred codons indicate the level of constraint due to splicing regulation. A distance effect (i.e., fewer unpreferred codons in ASEs further from exon 5) was detected in 2 amino acid residues (see Additional file 5).

A cline in base composition (i.e., GC-bias) along the exon cluster could explain the observed distance effect on the synonymous sites in these two codons. However, there was no correlation between the intron GC-content and the distance from exon 5 (*r *= -0.26, *P *= 0.08, Spearman's rank order correlation; *N *= 48). Taken together, these two codons in exon 6 ASEs are likely to be among the putative ESEs that control the distance effect. Although shared ancestry may violate the independence of the analyses, the usage patterns of the same two codons in other *Drosophila *species are largely similar to that in *D. melanogaster *(see Additional file 6).

### Synonymous divergence in the 3' boundary region and the remaining region is similar

**Figure 4:**
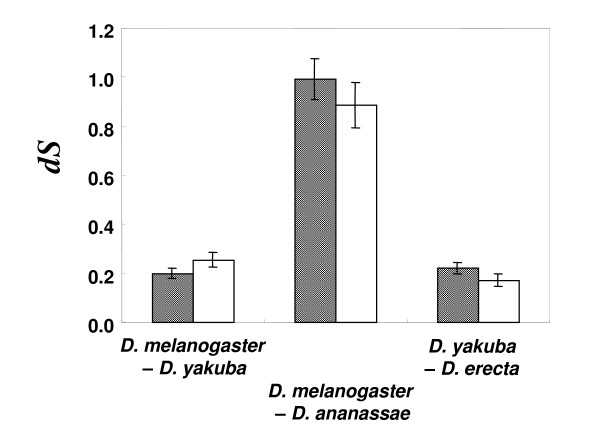
**Comparisons of synonymous divergence**. Synonymous divergence (*dS*) between 3 pairs of species estimated from the 3' boundary region and the remaining exonic region (center + 5' boundary), indicated by open and shaded bars, respectively. *dS *was estimated from the concatenated sequences of the same 24 orthologous pairs of ASEs that were identified in all five species pairs. Error bars represent standard errors estimated by the bootstrap method of 500 replications. No statistically significant difference between the two regions was observed in any of the species pairs.

The negative correlation observed between codon usage bias and synonymous divergence [8,46] suggests stronger purifying selection for translationally superior codons in highly biased genes. If there is purifying selection for ESE organization, lower synonymous divergence may be seen in the 3' boundary regions where the effect of splicing regulation is detected. To investigate this, I calculated synonymous divergence (*dS*) between different *Drosophila *species in the 3' boundary region and the remaining exonic region of exon 6 ASEs, whose regional differences in CBI appeared to be most apparent (Figure 2). The synonymous divergences between the two regions using the concatenated sequences of 24 orthologous ASE pairs were not significantly different in *D. melanogaster *– *D. yakuba*, *D. yakuba *– *D. erecta*, and *D. melanogaster *– *D. ananassae *species-pair comparisons (Figure 4). Similar patterns were also obtained for the constitutive exons of this gene (data not shown). Although the independence of these tests are violated by overlapping branches shared by these species-pairs, the data indicate that there is no apparent reduction of synonymous divergence due to purifying selection for splicing regulation.

The known ESEs in human and other species show degenerative features [18,44,47-49], which could be the reason for not observing strong purifying selection for particular codons involved in ESEs. However, Parmley et al. [50] showed that synonymous sites in putative ESE hexamers evolve more slowly than the remaining exonic sequences in mammalian genes. Although I have not specifically examined the difference between putative ESE sequences and other exonic sequences, the seeming inconsistency between mammalians and *Drosophila *may arise from the fact that there is no apparent codon bias for translational efficiency in the former. In *Drosophila*, it is the balance between the intensity of selection for translational efficiency and that for splicing efficiency that determines the relative rate of synonymous site substitutions. Therefore, the absence of apparent reduction of synonymous divergence in the 3' boundary regions compared to the remaining exonic regions suggests that the intensity of splice-related selection is generally weak and comparable to that for translational selection, which is known to be nearly neutral [16,51].

### Generality of the effect of exonic splicing regulation on synonymous codon usage

The genome-wide phenomenon of reduction of codon bias towards translationally superior codons in intron-exon boundary regions [20] was detected within the ASEs of exon 6 and 9 in *D. melanogaster*. The strong reduction in the 3' boundary regions in these exons indicates the presence of exonic signals involved in splicing regulation in these regions. I also examined this feature in other *Drosophila *species with similar codon bias patterns (see Additional files 7 and 8). The codon usage patterns of the 12 *Drosophila *species sequenced to date are similar to those of *D. melanogaster*, except for *D. willistoni *which has different usage patterns in some codons [36,52,53]. Since the codon abundance patterns in 5' and 3' ends of internal exons are highly correlated even between mouse and *Drosophila *[20], it can be assumed that the splice optimum codons are also mostly similar in these species. The tables in Additional files 7 and 8 indicate significant reductions in most of the species of CBI in the 3' boundary regions of exon 6 and 9 ASEs, respectively. Again, the shared ancestry violates independence, these data suggest that selection for splicing efficiency in the 3' regions may be a prominent factor affecting codon usage in these ASEs.

Is it possible that the reduction of the 3' boundary region compared to the remaining 5' region in exon 6 and 9 ASEs is due to the relaxation of purifying selection for translational optimum codons *per se*? An effect of the relaxed purifying selection is not seen in the synonymous divergence data (Figure 4). It is intuitively difficult to imagine a particular reason for relaxed selection for translational speed in partial exonic regions. Moreover, selection for translational accuracy favors translation with lower misincorporation rates of codons for functionally important amino acid residues, and thus, conserved amino acid residues should be more highly biased than others [10]. In my data, although the amino acid sequences are conserved in the 3' boundary region [21], the CBI values are reduced. This observation that the CBI values are reduced in the relatively conserved sequence regions is not consistent with the pattern expected by relaxed translational selection *per se *in the 3' boundary regions of these *Dscam *ASEs.

Another sensitive comparison using paralogous exonic regions to delineate the effect of splicing regulation on the coding exons was performed. Processed genes that have retrotransposed recently are copies of their parental genes without introns. These pairs of processed and parental genes enable comparisons between splice-present and splice-absent exonic regions with similar amino acid sequences. Betran et al. [54] have listed 24 young retroposed genes and their parental genes (more than 70% amino acid identity) found in the genome of *D. melanogaster*. 13 internal coding exons from the parental genes that were longer than 135 bp (or 45 full codons) were subjected to the following analyses (see Additional file 9).

**Figure 5:**
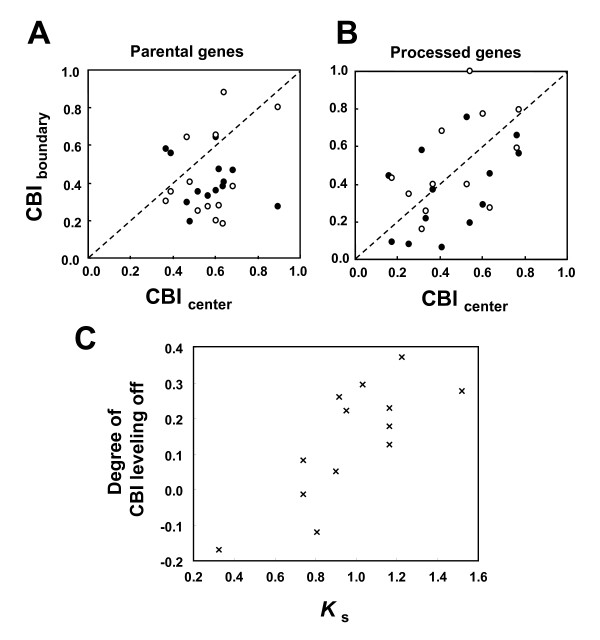
**Comparisons of processed genes and their parental genes**. Comparisons of internal coding exons longer than 45 full codons in the parental gene and their paralogous exonic regions in the intronless processed genes in *D. melanogaster*. Relationship between codon bias index (CBI) of the center and the intron-exon boundary regions (15 full codons from the junction) among the internal exons of the parental gene (A), and their corresponding exonic regions in the processed genes (B). Closed and open circles indicate values of 5' and 3' boundary regions, respectively. Relationship between *K*_s _estimated from the whole gene [54] and the degree of CBI leveling off, namely [Δ-CBI_central-boundary_]_parental gene _– [Δ-CBI_central-boundary_]_processed gene _(C). A positive correlation was observed between these values (*r *= 0.75, *P *< 0.01, Spearman's rank order correlation; *N *= 13).

CBI in the boundary regions were plotted against those in the central regions (Figure 5A, B), to determine whether CBI in the intron-exon boundary regions are reduced in the parental genes. In the parental genes, the differences are significant between the center and the 3' boundary (*z *= -2.13, *P *< 0.05, Wilcoxon matched-pair signed rank test; the same below, except where indicated) and between center and 5' boundary (*z *= -2.48, *P *< 0.05). This indicates that most of the intron-exon boundary regions in these internal exons have reduced CBI values, as shown in the whole genome analyses [20]. Next, the corresponding paralogous regions in the processed genes were examined. In the processed genes, the difference between the corresponding regions of the center and 3' boundary, and between the center and 5' boundary, were both not significant (*z *= 0.80, *P *= 0.42 and *z *= -1.29, *P *= 0.20, respectively). This means that after intronless retrotransposed copies land onto different genomic locations, the differences between what were formerly the central regions and the boundary regions becomes ambiguous. This leveling off should be proportional to the time since retroposition. The degree of leveling off was assessed by plotting the difference in Δ-CBI_central-boundary _between the parental and processed genes (see Methods) against *K*_s _of the whole gene (taken from [54]) in Figure 5C. A significant relationship between these two values was detected (*r *= 0.75, *P *< 0.01, Spearman's rank order correlation; *N *= 13), indicating leveling off of codon usage after the constraint has been removed.

Previously, Parmley et al. [55] have employed intronless retrogenes in mammals to examine the loss of selective constraints near intron-exon junctions. They observed higher rates of amino acid evolution near the domains where the intron-exon junctions previously resided, which indicates relaxation of constraints that existed in those boundary regions. The comparison with the parental genes allows delineation of the course of leveling off of differences in codon bias over time in the processed genes. This leveling off is a direct prediction of the trade-off model that invokes conflict between translational superiority and splicing regulation near the intron-exon boundary regions [20]. Thus, these results corroborate the generality of the effect of splicing regulation on synonymous codon usage which is observed among genome-wide genes in *Drosophila *[20], and supports the conclusions obtained from ASEs of *Dscam*.

## Conclusion

In the conventional framework of codon usage bias towards translationally superior codons in *Drosophila*, I was able to detect a counteracting selective force of splicing regulation in the 3' intron-exon boundary regions of the *Dscam *exon 6 and 9 ASEs. The positive correlations detected between the expression level (splicing frequency) and Δ-CBI_rest – 3' boundary _are clear evidence that synonymous codon usage is restricted by exonic splicing signals in these ASEs. This is the first study that directly compare splicing frequency with codon usage. Furthermore, I have shown that two of the codons in exon 6 ASEs may play a role in controlling the distance effect between splice sites, possibly through changing the strength of potential ESEs by altering codon usage. A similar synonymous divergence level between different exonic regions suggests that the intensity of splice-related selection is generally weak and comparable to that of translational selection. Taken together, these results demonstrate that *Dscam *provides one of the best-controlled and thus sensitive systems to study the effects of such splice-related selection. In addition, the analyses on the processed genes and their parental genes delineate the generality of the effect of splicing regulation on synonymous codon usage, which is another concrete example of weak natural selection.

## Methods

### Sequence Data

The sequence deposited as accession #AF260530 for *D. melanogaster Dscam *was used. For other species, sequences from the Comparative Assembly Freeze 1 (CAF1) of Assembly/Alignment/Annotation of 12 related Drosophila species  were manually annotated for all the putative ASEs. These initially annotated sequences were checked for any updates in the Flybase Release FB_10 on Nov. 19, 2008. Sequences with internal stop codons and those lacking the conventional AG/GT intron splice sites were excluded from the analyses. Thus, some of the ASEs could have been missed due to minor errors in sequence assembly.

### Calculation of Codon Bias Index (CBI)

The Codon Bias Index (CBI) is a measure of the fraction of codons biased towards preferred triplets [6]. A value of 1 indicates that only preferred codons were used in all of the triplets in the mRNA. A value of 0 indicates totally random choice. Negative values are possible when unpreferred codons are used more than expected. CBI of each exonic region was calculated using codonW by J. Peden . CBI of *D. melanogaster *were calculated using the preferred and unpreferred codon table implemented in the program. The *D. simulans *preferred and unpreferred codon usage table [56] were used for calculation of CBI in *D. simulans*, *D. sechellia*, *D. yakuba*, and *D. erecta*. CBI were calculated for *D. ananassae*, *D. pseudoobscura*, *D. persimilis*, *D. virilis*, *D. mojavensis*, and *D. grimshawi *using the *D. pseudoobscura *preferred and unpreferred codon usage table [56]. *D. willistoni *was excluded from the analyses due to its weak codon usage bias and shifted pattern of preferred codons for a portion of amino acids [36,52,53].

### Comparison of CBI between constitutive and alternatively spliced exons

CBI of whole exonic regions in the 17 internal constitutive exons without UTRs (exon 3, 5, 7, 8, 10, 11, 12, 13, 14, 15, 16, 18, 19, 20, 21, 22, and 23) and of 95 ASEs (exon 4.1–4.12, 6.1–6.48, 9.1–9.33, 17.1–17.2) were calculated as above. Average CBI of both constitutive and alternatively spliced exons were calculated and compared by simple Student's *t*-test. The result should be interpreted with caution due to possible non-independence of the pooled ASE data from different exon clusters.

### Splicing frequency data

The relative orders of splicing frequency of the exon 6 and 9 ASEs were obtained from the expression level data of hemocyte-derived S2 cell lines by Neves et al. [43]. The splicing-frequency order of exon 6 ASEs was taken from their Supplementary Figure One b, where they showed the over-representation levels of ASEs in the total *Dscam *mRNA population amplified by PCR primers designed within exon 3 and exon 10 (constitutive exons). Relative levels of ASE use in those S2 cells were similar, with some moderate differences to levels in hemocytes and neurons of third instar larvae, and to levels in whole individuals from different developmental stages [43]. In the case of exon 9, the relative order of the expression level was taken from Figure Four a of Neves et al. [43], which correlates well with the splicing-frequency order in hemocytes of third instar larvae and with that in whole individuals from embryonic and adult stages. In my analyses, expression levels in S2 cell lines were used because those cells represent a simple *in vitro *system in which this molecule is transcribed and processed autonomously.

### Calculation of synonymous divergence

Calculation of synonymous divergence (*dS*) was conducted using *MEGA *version 3.1 [57]. *dS *was estimated by modified Nei-Gojobori method with Jukes-Canter corrections for multiple-hits. Transition/transversion ratio of 2 was assumed. Standard errors were estimated using the bootstrap method with 500 replications as implemented in the program.

Orthologous pairs of ASEs were chosen from the neighbor-joining tree using amino acid *p*-distance. Only one to one orthologies supported by higher than 80% bootstrap values were accepted. 24 ASEs whose orthologous pairs were found between all 3 species pairs (*D. melanogaster *– *D. yakuba*, *D. melanogaster *– *D. ananassae*, and *D. yakuba *– *D. erecta*) were used for the calculation of synonymous divergence. Those ASEs were exon 6.1–6.5, 6.7, 6.10, 6.12, 6.13, 6.15–6.18, 6.23, 6.24, 6.28, 6.30–6.33, 6.36, and 6.45–6.47 in *D. melanogaster*. The 3' intron-exon boundary regions (45 bp) and the remaining exonic regions of these ASEs were concatenated separately before calculating divergence.

### Data used for the processed genes versus parental gene comparison

24 young retroposed genes in the genome of *D. melanogaster *were identified by Betran et al. [54]. Among the exons of the parental genes of these young genes, the criteria were set to choose internal coding exons longer than 135 bp (or 45 full codons). There were 13 such exons from 11 parental genes (see Additional file 9). CBI of the center and the boundary regions were calculated from these exons and the corresponding exonic regions of the processed genes.

### Calculation of the degree of CBI leveling off

The degree of CBI leveling off was estimated by the difference in Δ-CBI_central-boundary _= CBI_central _– (CBI_5'boundary _+ CBI_3'boundary_)/2 between the internal exons in the parental genes and their paralogous exonic regions in the intronless processed genes. This difference, [Δ-CBI_central-boundary_]_parental gene _– [Δ-CBI_central-boundary_]_processed gene_, increases as the reduction of CBI in the intron-exon boundary regions becomes small in the processed genes.

## Authors' contributions

AT conceived of the study, performed the statistical analyses, and wrote the manuscript. The final manuscript was read and approved by AT.

## Supplementary Material

Additional file 1 **Codon usage in each amino acid residue**. Regional differences in frequencies of translationally preferred codons [58] used for each degenerative codons among the ASEs of *Dscam *exons 6 and 9 in *D. melanogaster*. Open bars indicate frequencies in the 3' intron-exon boundary region and shaded bars indicate those in the remaining exonic region. Numbers above the bars indicate observed numbers of amino acids. ** indicates P < 0.001 by Fisher's exact test after Bonferronni correction. * indicates P < 0.05 without correction. Click here for file

Additional file 2 **Trends in SMA**. Relationship between expression level and CBI in the 3' intron-exon boundary region (A, B) and that between expression level and the ratio of the "frequency of preferred codons near the boundary/frequency of avoided codons near the boundary" in the 3' intron-exon boundary region (C, D) in *D. melanogaster Dscam *exon 6 ASEs. Preferred and avoided codons near the intron-exon boundary regions were taken from Warnecke and Hurst [20]. Simple moving average (SMA) of 5 (A, C) and 10 (B, D) datapoints by expression level order after removing one outlier each (larger than 3 × S. D.) are plotted against their midpoint order. Correlation coefficients (*r*) by Spearman's rank order correlation are shown above the graphs. Click here for file

Additional file 3 **Relationship between CBI and expression level in ASEs of exon 9**. Relationship between codon usage and the relative order of expression level (splicing frequency) of the *D. melanogaster Dscam *exon 9 ASEs in hemocyte-derived S2 cell lines [43]. Relationship between expression level and the difference in CBI between the 5' intron-exon boundary plus central regions and the 3' intron-exon boundary regions (Δ-CBI_rest – 3'boundary_; A), that between expression level and CBI in the 5' intron-exon boundary plus central regions (CBI_5'boundary + central_; B), and that between expression level and CBI in the 3' intron-exon boundary regions (CBI_3'boundary_; C). Only the last half of the ASE s ordered from the lowest to the highest expression level (orders 17 – 32) was used for the analyses. The average and the S. E. value of the rest of the ASEs (orders 1–16) are shown at the left end of each graph. Click here for file

Additional file 4 **Positions of the conserved amino acid residues**. Positions of the 4 conserved amino acid residues in the 3' boundary region among all the 48 ASEs of *D. melanogaster Dscam *exon 6. Click here for file

Additional file 5 **The effect of nucleotide distance on codon usage**. Numbers of preferred and unpreferred codons used in the conserved amino acid residues among *D. melanogaster Dscam *exon 6 ASEs. Codons were divided into 3 groups by the proximity to exon 5. Click here for file

Additional file 6 **Codon usage in conserved amino acid residues in the ASEs of exon 6**. Frequencies of the translationally unpreferred codons used in the conserved amino acid residues of all 48 ASEs of *Dscam *exon 6. Colors represent codon usages in 11 *Drosophila *species. Numbers 1, 2, and 3 in the X-axis indicate ASEs positioned closest, intermediate, and farthest to exon 5, respectively, when categorized into 3 equal numbered groups. Click here for file

Additional file 7 **CBI values of exon 6 ASEs in other *Drosophila *species**. Comparison of the CBI values between the center and the boundary regions of *Dscam *exon 6 ASEs in other *Drosophila *species. Click here for file

Additional file 8 **CBI values of exon 9 ASEs in other Drosophila species**. Comparison of the CBI values between the center and the boundary regions of *Dscam *exon 9 ASEs in other *Drosophila *species. Click here for file

Additional file 9 **List of processed and parental genes**. Codon Bias Index (CBI) of each exonic region of internal coding exons longer than 135 bp in the parental genes and their paralogous exonic regions in the processed genes in *D. melanogaster *chosen from Table One of Betran et al. [54]. Click here for file
