# Supplementary figures and images for: Effect of exonic splicing regulation on synonymous codon usage in alternatively spliced exons of Dscam
Source: BMC Evol Biol. 2009 Aug 27;9:214. doi: 10.1186/1471-2148-9-214 (PMC2741454; doi:10.1186/1471-2148-9-214)

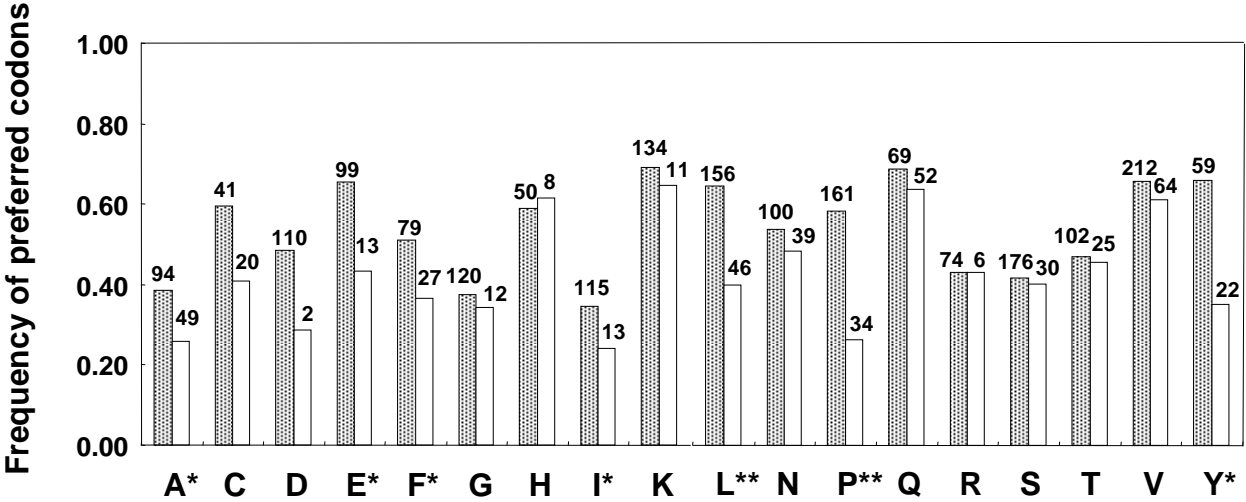

Supplement: Additional file 1 — Codon usage in each amino acid residue. Regional differences in frequencies of translationally preferred codons [58] used for each degenerative codons among the ASEs of Dscam exons 6 and 9 in D. melanogaster. Open bars indicate frequencies in the 3' intron-exon boundary region and shaded bars indicate those in the remaining exonic region. Numbers above the bars indicate observed numbers of amino acids. ** indicates P < 0.001 by Fisher's exact test after Bonferronni correction. * indicates P < 0.05 without correction. [file 1471-2148-9-214-S1.pdf]

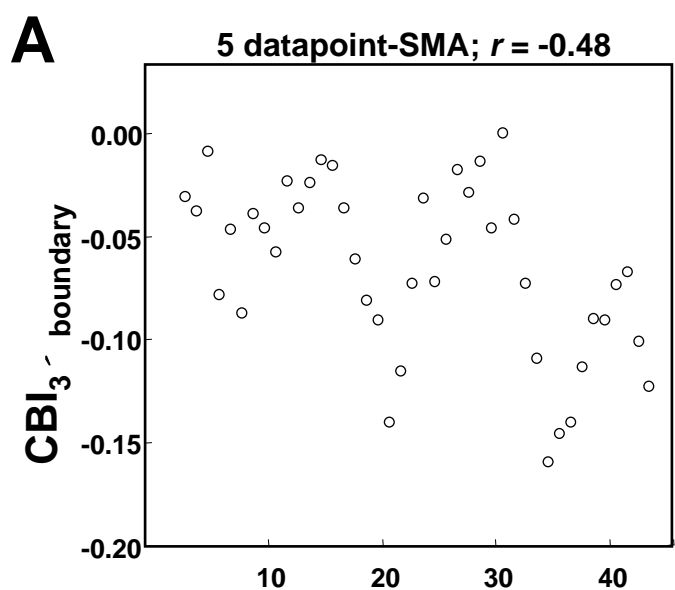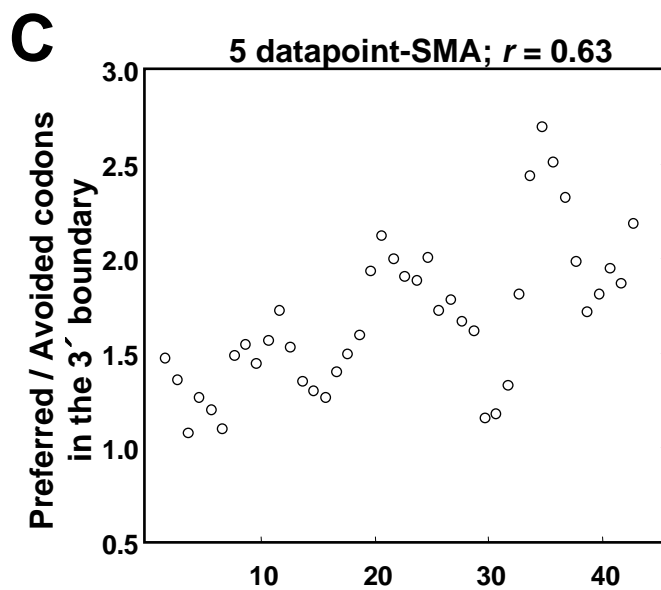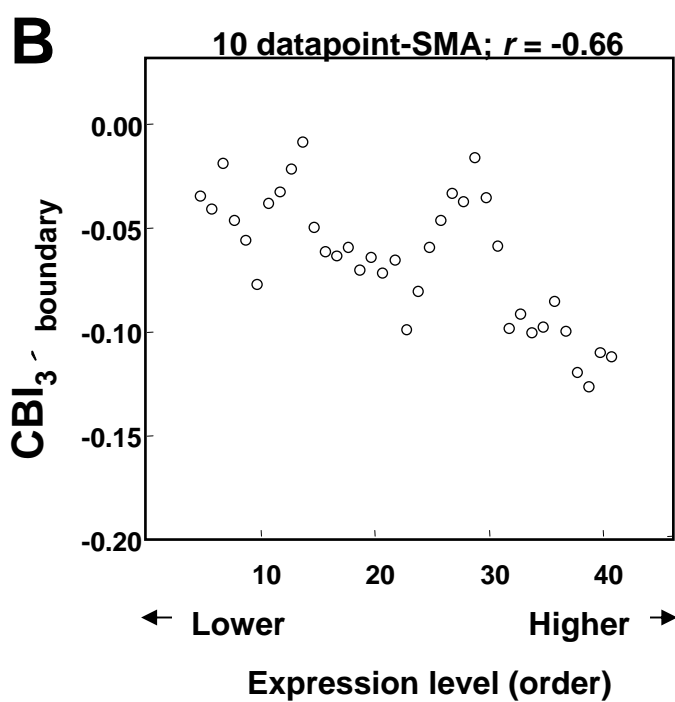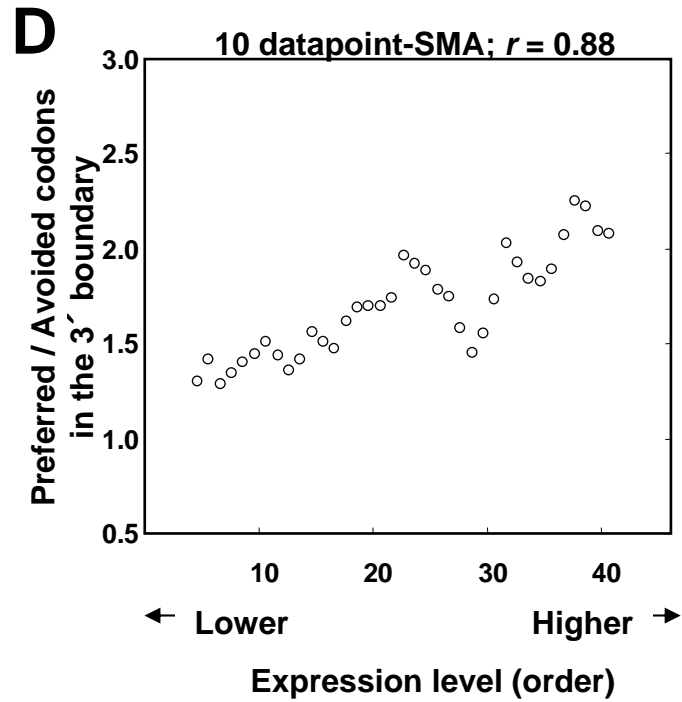

Supplement: Additional file 2 — Trends in SMA. Relationship between expression level and CBI in the 3' intron-exon boundary region (A, B) and that between expression level and the ratio of the "frequency of preferred codons near the boundary/frequency of avoided codons near the boundary" in the 3' intron-exon boundary region (C, D) in D. melanogaster Dscam exon 6 ASEs. Preferred and avoided codons near the intron-exon boundary regions were taken from Warnecke and Hurst [20]. Simple moving average (SMA) of 5 (A, C) and 10 (B, D) datapoints by expression level order after removing one outlier each (larger than 3 × S. D.) are plotted against their midpoint order. Correlation coefficients (r) by Spearman's rank order correlation are shown above the graphs. [file 1471-2148-9-214-S2.pdf]

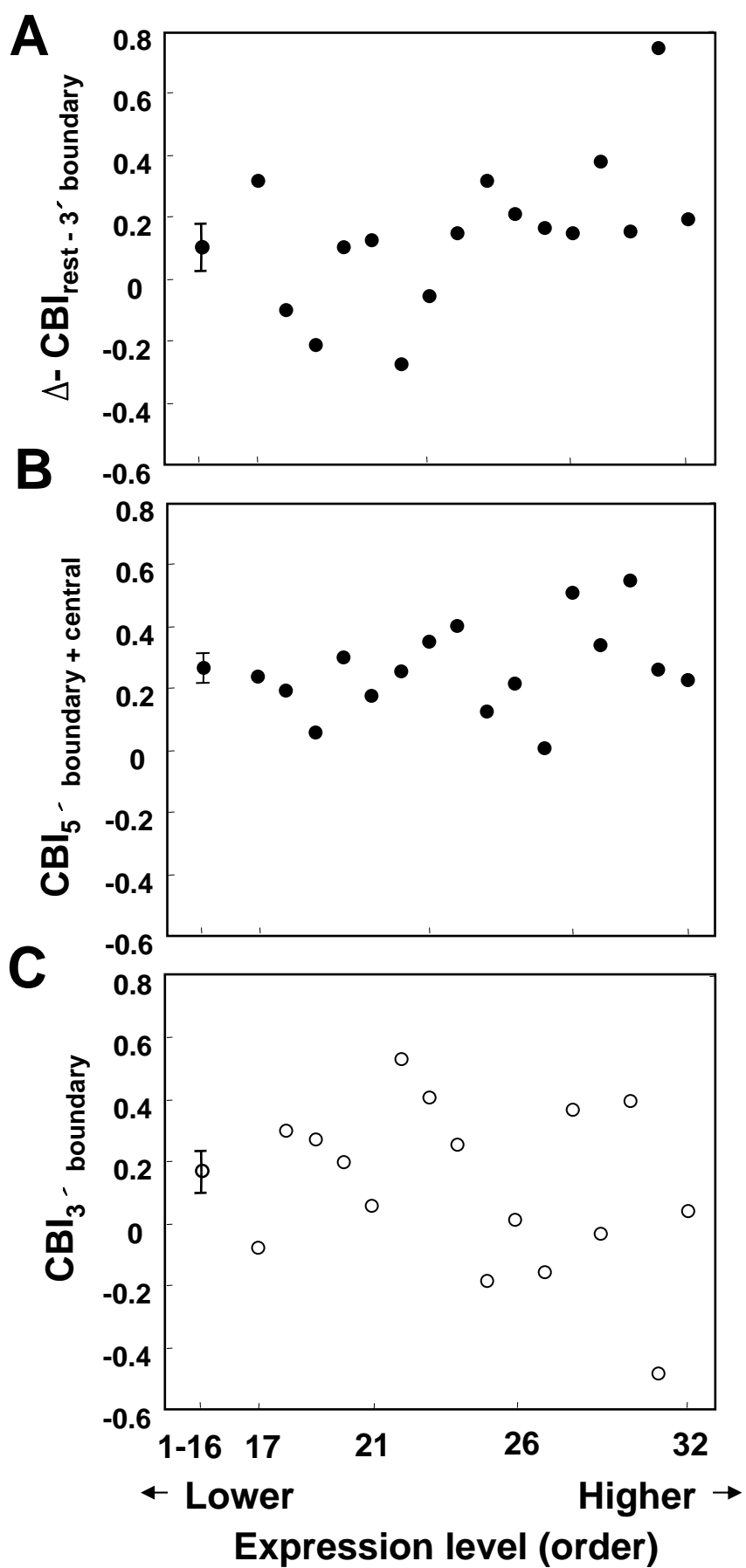

Supplement: Additional file 3 — Relationship between CBI and expression level in ASEs of exon 9. Relationship between codon usage and the relative order of expression level (splicing frequency) of the D. melanogaster Dscam exon 9 ASEs in hemocyte-derived S2 cell lines [43]. Relationship between expression level and the difference in CBI between the 5' intron-exon boundary plus central regions and the 3' intron-exon boundary regions (Δ-CBIrest – 3'boundary; A), that between expression level and CBI in the 5' intron-exon boundary plus central regions (CBI5'boundary + central; B), and that between expression level and CBI in the 3' intron-exon boundary regions (CBI3'boundary; C). Only the last half of the ASE s ordered from the lowest to the highest expression level (orders 17 – 32) was used for the analyses. The average and the S. E. value of the rest of the ASEs (orders 1–16) are shown at the left end of each graph. [file 1471-2148-9-214-S3.pdf]

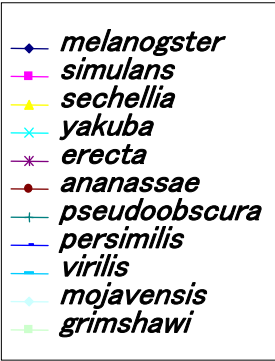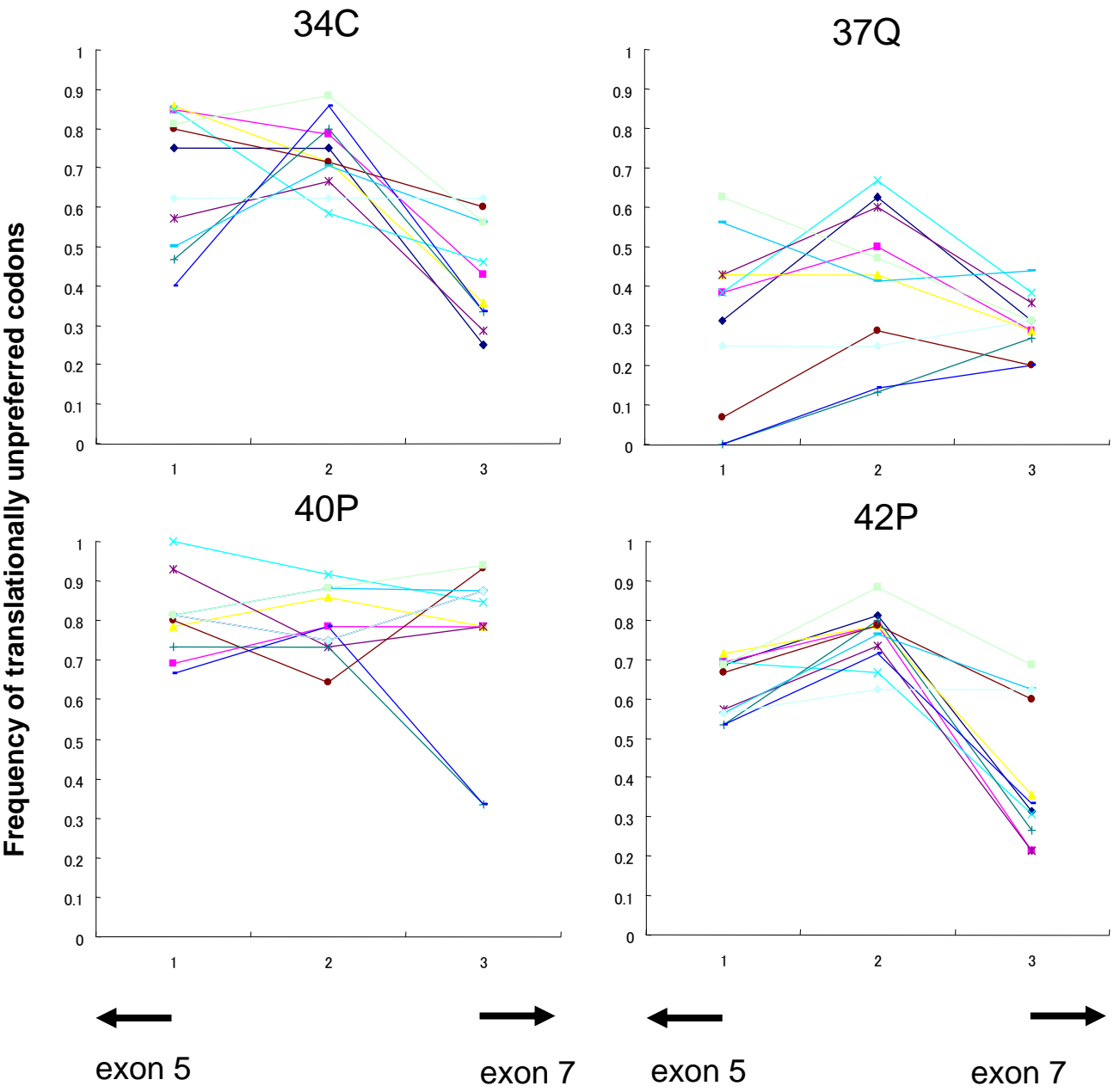

Supplement: Additional file 6 — Codon usage in conserved amino acid residues in the ASEs of exon 6. Frequencies of the translationally unpreferred codons used in the conserved amino acid residues of all 48 ASEs of Dscam exon 6. Colors represent codon usages in 11 Drosophila species. Numbers 1, 2, and 3 in the X-axis indicate ASEs positioned closest, intermediate, and farthest to exon 5, respectively, when categorized into 3 equal numbered groups. [file 1471-2148-9-214-S6.pdf]
