# Supplementary material for: Effect of exonic splicing regulation on synonymous codon usage in alternatively spliced exons of Dscam
Source: BMC Evol Biol. 2009 Aug 27;9:214. doi: 10.1186/1471-2148-9-214 (PMC2741454; doi:10.1186/1471-2148-9-214)
Supplement: Additional file 4 — Positions of the conserved amino acid residues. Positions of the 4 conserved amino acid residues in the 3' boundary region among all the 48 ASEs of D. melanogaster Dscam exon 6. [file 1471-2148-9-214-S4.pdf]

Conserved AA position

34 37 40 42

```
Dscam_exon6.01    PVGRVSPKFP-NTLTSSSFTGD---EGSSQTLLCPAQAYPAPLF
Dscam_exon6.02    PIASVGPRLL-SGNDIKVLQFS---ASQASTLLCPAQSYPVPVF
Dscam_exon6.03    PVGSIGPRLT-SGDESRILRVY---LAASATLLCPAQAYPVPFF
Dscam_exon6.04    PVGSVSPQLS-GNGNQEHITLTRVPKMGSVTLMCPAQAYPVPFF
Dscam_exon6.05    PVGSVGPKLT-SGDDSRTVRIR---QEDSVTLLCPAQAYPVPVY
Dscam_exon6.06    PVGTVSPKIS-TGEDFKHLKGK---SSQDLSILCPAQAYPQPFF
Dscam_exon6.07    PIGSVSPKIS-NSDDLKHVKVK---RLLSLSLLCPAQAYPQPIS
Dscam_exon6.08    PVGSVAPKLN-FLERFKMMQIQ---SGTSFNIQCPVQSYPMPVF
Dscam_exon6.09    PVGSVRPKVN-PQDKHQFIDVE---LASSYSLLCMAQSYPIPSF
Dscam_exon6.10    PVGSVKPKIN-VQDKLQTREIS---QGIGIALLCPAQSYPIPAH
Dscam_exon6.11    HCCLSDYSIR-FIGMWFLSQLR-----PMIVLKCPSQTYPLPAC
Dscam_exon6.12    PVASVPPKLN-LLERVRTMEVS---VGTKYAMQCPGQAFPVPIN
Dscam_exon6.13    PIGSVPPKINNINDKFQLMQVK---LESSFAMQCPGQAYPVPVV
Dscam_exon6.14    PIGSVPPKITSLHDKFQVLQVK---LAEDFSMQCPGQAYPVPIV
Dscam_exon6.15    PVGSVAPKVD-TRDEFTFARTR---MGASKALICPAQSYPMPFF
Dscam_exon6.16    PVGSVAPKVD-IKDEINYARVG---QDRSLAIVCPAQSYPVPA
Dscam_exon6.17    PVGSVAPKVD-PNDRIKWVDKP---RGSSLNLLCPAQSFPMPSA
Dscam_exon6.18    PVGSVAPKVD-VKDRITWLDKP---MGQALSLLCPAQSYPMPVY
Dscam_exon6.19    PVGSVAPKVD-MHDKMDFTIRS---SNRSLNLFCKAQGFPMPAF
Dscam_exon6.20    PVGSVAPKVD-MQDKFGVNIRQ---ANNSLNLLCKAQGYPMPAF
Dscam_exon6.21    PVGSVGPKVE-IKDKVGIFVAK---SNSSLALLCPAQSYPIPAF
Dscam_exon6.22    PIGSVAPRVN-RKDEFNHDRIK---SSKTISIQCPAQAFPVPVY
Dscam_exon6.23    PVGSVAPKVN-KNDEFSHDRIA---IGGMVALRCPAQSFPVPVY
Dscam_exon6.24    PVGSVAPRVN-KKDGFSHDSIR---QDRTIAIFCPAQAYPVPFY
Dscam_exon6.25    PVGSVQPKIS-VGERRKDAEVP---KHGDLSIFCPAQSYPVPAF
Dscam_exon6.26    PVGSVGPKIS-VGERLRDAGAP---SKSTLTLFCPAQSYPVPSF
Dscam_exon6.27    PVGSVAPKIS-VENRLKNAEAR---IGSTFTLFCPGQAYPVPFF
Dscam_exon6.28    PVGSVGPRLT-SGDKTRNVDIS---LAGSGTLLCPAQAYPVPFF
Dscam_exon6.29    PISGAAPRV-ALIQKTNIEYVR---LGEIVSIMCPAQGFPVPAF
Dscam_exon6.30    PISSAVPKVVSLAKFD-MKTYS---GSSTMALLCPAQGYPVPVF
Dscam_exon6.31    PVSSTAPKVPPLQSRP--MIVP---GHTDLSILCPAQGYPAPSF
Dscam_exon6.32    PVSSSPPKINTLTYKP--NIVE---SMASTAILCPAQGYPAPSF
Dscam_exon6.33    PVGGKAPTLLSNDIKRSWIERM---LSTNLALFCPAQGFPVPAF
Dscam_exon6.34    PISSSAPRTPALVQKP--LELM---VAHTISLLCPAQGFPAPSF
Dscam_exon6.35    PISAKAPVLAV-DTKWSGIERH---RDMDIVLLCPAQAYPVPIS
Dscam_exon6.36    PVGAKAPTFSL-EYKLIEVQKS---KGTAFALLCQAQAFPVPII
Dscam_exon6.37    PIGSKAPSVPT-GVMVFQQQVP---LLSTFAMLCQAQAFPVPVF
Dscam_exon6.38    PIGSKAPAVPT-GVLAFQQNVP---IHSIFAMLCQAQAFPVPIY
Dscam_exon6.39    PVGFKAPSFPS-VASTFTIQSP---KKSELALTCEAQSYPVPIF
Dscam_exon6.40    PVGAKAPTFSS-DSKGSIFERS---IKASFALLCQAQAFPVPII
Dscam_exon6.41    PIGARAPSFSS-DYNSFSYARV---VDQSFAMLCQAQAFPLPIM
Dscam_exon6.42    PIGARAPTFST-DSNSFSYTRS---VGQSFALLCQAQAYPVPIM
Dscam_exon6.43    PIGARAPTFST-DSIVFSYIRP---VGQSFALLCQAQAYPVPAM
Dscam_exon6.44    PVGSKAPTFAT-ASKISSLLGS---SSSDIVLLCQAQAFPVPYT
Dscam_exon6.45    PVSAKGPTFAS-DMKSYTFVRH---FGQSFALLCQAQAYPVPLI
Dscam_exon6.46    PVGAKAPTFSG-ESKSFTFVKE---ISTSFALLCQAQAFPVPII
Dscam_exon6.47    PVGSVSPKIS--GGRLQEITKS---AATDLAILCLGQSFPIPAF
Dscam_exon6.48    PIGSVAPTIS--GDRLQEQTRK---LGHSFSILCQGQAYPHPVF
```

Additional file 4
