# Supplementary material for: Effect of exonic splicing regulation on synonymous codon usage in alternatively spliced exons of Dscam
Source: BMC Evol Biol. 2009 Aug 27;9:214. doi: 10.1186/1471-2148-9-214 (PMC2741454; doi:10.1186/1471-2148-9-214)
Supplement: Additional file 5 — The effect of nucleotide distance on codon usage. Numbers of preferred and unpreferred codons used in the conserved amino acid residues among D. melanogaster Dscam exon 6 ASEs. Codons were divided into 3 groups by the proximity to exon 5. [file 1471-2148-9-214-S5.pdf]

Additional file 5

Conserved amino acid residue	C at position 34		Q at position 37		P at position 40		P at position 42	
Codon usage preference for translational efficiency [a]	Pref. UGC	Unpref. UGU	Pref. CAG	Unpref. CAA	Pref. CCC	Unpref. CCU CCG CCA	Pref. CCC	Unpref. CCU CCG CCA
Number of codons used								
Dscam exon 6.1 – 6.16 (N = 16)	4	12	11	5	3	13	5	11
Dscam exon 6.17 – 6.32 (N = 16)	4	12	6	10	4	12	3	13
Dscam exon 6.33 – 6.48 (N = 16)	12	4	11	5	2	14	11	5
Total	20	< 28	28	> 20	9	< 39	19	< 29
Fisher's exact test (3x2) [b]	P <0.05		n.s.		n.s.		P<0.05	

[a] Preferred codons based on Sharp and Lloyd [58]

[b] Bonferroni correction applied.
