# Supplementary material for: Effect of exonic splicing regulation on synonymous codon usage in alternatively spliced exons of Dscam
Source: BMC Evol Biol. 2009 Aug 27;9:214. doi: 10.1186/1471-2148-9-214 (PMC2741454; doi:10.1186/1471-2148-9-214)
Supplement: Additional file 7 — CBI values of exon 6 ASEs in other Drosophila species. Comparison of the CBI values between the center and the boundary regions of Dscam exon 6 ASEs in other Drosophila species. [file 1471-2148-9-214-S7.pdf]

Additional file 7

	N	CBI$_{center}$ vs. CBI$_{3' boundary}$			CBI$_{center}$ vs. CBI$_{5' boundary}$			CBI$_{5' boundary}$ vs. CBI$_{3' boundary}$		
		T^a	z	P-value[d]	T^b	z	P-value[d]	T^c	z	P-value[d]
D. simulans	41	87	-4.451	****	257	-2.248	n.s.[e]	111	-4.140	***
D. sechellia	42	104	-4.345	****	283	-2.107	n.s.[e]	106	-4.320	****
D. yakuba	38	122	-3.604	**	290	-1.167	n.s.	134	-3.430	**
D. erecta	43	155	-3.840	**	335	-1.666	n.s.	180	-3.538	**
D. ananassae	44	273	-2.591	n.s.[e]	595	1.167	n.s.	133	-4.225	***
D. pseudoobscura	45	419	-1.112	n.s.	611	1.055	n.s.	320	-2.229	n.s.[e]
D. persimilis	44	427	-0.794	n.s.	609	1.330	n.s.	308	-2.182	n.s.[e]
D. virilis	49	384	-2.273	n.s.[e]	620	0.075	n.s.	339	-2.721	*
D. mojavensis	48	289	-3.067	*	751	1.672	n.s.[e]	175	-4.236	***
D. grimshawi	52	360	-2.996	*	845	1.421	n.s.	194	-4.508	****

[a] Absolute value of the sum of negative ranks of the difference in CBI (CBI$_{center}$ – CBI$_{3' boundary}$).

[b] Absolute value of the sum of negative ranks of the difference in CBI (CBI$_{center}$ – CBI$_{5' boundary}$).

[c] Absolute value of the sum of negative ranks of the difference in CBI ($CBI_{5'\ \mathrm{boundary}} - CBI_{3'\ \mathrm{boundary}}$).

[d] * P<0.05, ** P<0.01, *** P<0.001, **** P<0.0001, by Wilcoxon’s rank sum test after Bonferroni correction for multiple (10) tests.

[e] P<0.05 by Wilcoxon’s rank sum test before correction.
