# Supplementary material for: Effect of exonic splicing regulation on synonymous codon usage in alternatively spliced exons of Dscam
Source: BMC Evol Biol. 2009 Aug 27;9:214. doi: 10.1186/1471-2148-9-214 (PMC2741454; doi:10.1186/1471-2148-9-214)
Supplement: Additional file 8 — CBI values of exon 9 ASEs in other Drosophila species. Comparison of the CBI values between the center and the boundary regions of Dscam exon 9 ASEs in other Drosophila species. [file 1471-2148-9-214-S8.pdf]

Additional file 8

	N	CBI$_{center}$ vs. CBI$_{3^{\prime} boundary}$			CBI$_{center}$ vs. CBI$_{5^{\prime} boundary}$			CBI$_{5^{\prime} boundary}$ vs. CBI$_{3^{\prime} boundary}$		
		T[a]	*z*	*P*-value[d]	*T*[b]	*z*	*P*-value[d]	*T*[c]	*z*	*P*-value[d]
D. simulans	-	-	-	-	-	-	-	-	-	-
D. sechellia	31	134	-2.234	n.s.[e]	228	-0.392	n.s.	173	-1.47	n.s.
D. yakuba	32	137	-2.375	n.s.[e]	259	-0.093	n.s.	166	-1.832	n.s.
D. erecta	32	97	-3.123	*	238	-0.486	n.s.	149	-2.15	n.s.[e]
D. ananassae	32	98	-3.104	*	192	-1.346	n.s.	200	-1.197	n.s.
D. pseudoobscura	32	55	-3.908	***	162	-1.907	n.s.	134	-2.431	n.s.[e]
D. persimilis	29	97	-2.606	n.s.[e]	66	-3.276	**	231	0.292	n.s.
D. virilis	32	58	-3.852	***	134	-2.431	n.s.[e]	177	-1.627	n.s.
D. mojavensis	31	34	-4.194	***	61	-3.665	**	173	-1.47	n.s.
D. grimshawi	32	53	-3.945	***	186	-1.459	n.s.	123	-2.637	n.s.[e]

[a] Absolute value of the sum of negative ranks of the difference in CBI (CBI$_{center}$ – CBI$_{3^{\prime} boundary}$).

[b] Absolute value of the sum of negative ranks of the difference in CBI (CBI$_{center}$ – CBI$_{5^{\prime} boundary}$).

[c] Absolute value of the sum of negative ranks of the difference in CBI ($\mathrm{CBI}_{5'\ \mathrm{boundary}} - \mathrm{CBI}_{3'\ \mathrm{boundary}}$).

[d] * P<0.05, ** P<0.01, *** P<0.001, **** P<0.0001, by Wilcoxon’s rank sum test after Bonferroni correction for multiple (10) tests.

[e] P<0.05 by Wilcoxon’s rank sum test before correction.
