# Supplementary material for: Effect of exonic splicing regulation on synonymous codon usage in alternatively spliced exons of Dscam
Source: BMC Evol Biol. 2009 Aug 27;9:214. doi: 10.1186/1471-2148-9-214 (PMC2741454; doi:10.1186/1471-2148-9-214)
Supplement: Additional file 9 — List of processed and parental genes. Codon Bias Index (CBI) of each exonic region of internal coding exons longer than 135 bp in the parental genes and their paralogous exonic regions in the processed genes in D. melanogaster chosen from Table One of Betran et al. [54]. [file 1471-2148-9-214-S9.pdf]

Additional file 9

#	Parental genes: Locus	internal exons (length in numbers of full codons)	$CBI_{central}$	$CBI_{5' boudary}$	$CBI_{3' boudary}$	Processed genes: Locus	$CBI_{central}$	$CBI_{5' boudary}$	$CBI_{3' boudary}$	K_S [a] whole gene
1	*CG8331*	exon 2 (77)	0.367	0.579	0.300	*CG4960*	0.333	0.215	0.257	0.32681
2	*CG17734*	exon 2 (62)	0.466	0.294	0.644	*CG11825*	0.174	0.092	0.429	0.73976
3	*Atg8a*	exon 2 (66)	0.683	0.464	0.379	*CG12334*	0.636	0.455	0.270	0.73999
4	*CG8310*	exon 3 (121)	0.604	0.644	0.654	*Vha36*	0.604	0.287	0.774	0.80580
5	*Trxr1*	exon 2 (114)	0.642	0.405	0.881	*Trxr2*	0.543	0.193	1.000	0.89926
6	*Ef1alpha100E*	exon 2 (68)	0.635	0.381	0.179	*Ef1alpha 48D*	0.774	0.564	0.797	0.91664
7	*Pros28.1*	exon 2 (76)	0.392	0.556	0.351	*Pros28.1A*	0.160	0.444	0.444	0.95103
8	*CanB2*	exon 3 (99)	0.619	0.474	0.280	*CanB*	0.315	0.579	0.160	1.03241
9	*CanA1*	exon 2 (110)	0.520	0.351	0.250	*CG9819*	0.411	0.061	0.679	1.16483
10		exon 4 (48)	0.482	0.192	0.405		0.528	0.755	0.395	
11		exon 5 (72)	0.564	0.327	0.270		0.762	0.657	0.590	
12	*CG9091*	exon 2 (45)	0.897	0.270	0.803	*CG9873*	0.368	0.368	0.395	1.22348
13	*Acon*	exon 3 (168)	0.602	0.357	0.200	*CG4706*	0.257	0.077	0.345	1.52027

[a] K_S values from the whole gene comparison were taken from Betran *et al*. [54].
